# Involvement of Reactive Oxygen Species in ABA-Induced Increase in Hydraulic Conductivity and Aquaporin Abundance

**DOI:** 10.3390/ijms22179144

**Published:** 2021-08-24

**Authors:** Guzel Sharipova, Ruslan Ivanov, Dmitriy Veselov, Guzel Akhiyarova, Maria Shishova, Tatyana Nuzhnaya, Guzel Kudoyarova

**Affiliations:** 1 Ufa Institute of Biology of Ufa Federal Research Centre of the Russian Academy of Sciences, Pr. Octyabrya 69, 450054 Ufa, Russia; g.v.sharipova@mail.ru (G.S.); ivanovirs@mail.ru (R.I.); akhiyarova@rambler.ru (G.A.); tanyawww89@mail.ru (T.N.); guzel@anrb.ru (G.K.); 2 Department of Plant Physiology and Biochemistry, Faculty of Biology, Saint Petersburg State University, Universitetskaya nab. 7–9, 199034 St. Petersburg, Russia; mshishova@mail.ru; 3 Institute of Biochemistry and Genetics, Ufa Federal Research Centre, Russian Academy of Sciences, Prospekt Oktyabrya 71, 450054 Ufa, Russia

**Keywords:** ABA, aquaporins, ROS scavenger, ascorbic acid, diphenyleneiodonium chloride

## Abstract

The role of reactive oxygen species (ROS) in ABA-induced increase in hydraulic conductivity was hypothesized to be dependent on an increase in aquaporin water channel (AQP) abundance. Single ABA application or its combination with ROS manipulators (ROS scavenger ascorbic acid and NADPH oxidase inhibitor diphenyleneiodonium chloride (DPI)) were studied on detached roots of barley plants. We measured the osmotically driven flow rate of xylem sap and calculated root hydraulic conductivity. In parallel, immunolocalization of ABA and HvPIP2;2 AQPs was performed with corresponding specific antibodies. ABA treatment increased the flow rate of xylem, root hydraulic conductivity and immunostaining for ABA and HvPIP2;2, while the addition of antioxidants prevented the effects of this hormone. The obtained results confirmed the involvement of ROS in ABA effect on hydraulic conductivity, in particular, the importance of H_2_O_2_ production by ABA-treated plants for the effect of this hormone on AQP abundance.

## 1. Introduction

An accumulation of various forms of reactive oxygen species (ROS) is triggered by a wide range of factors, including hormonal signals [1] and environmental cues [2]. ABA-induced ROS elevation has been repeatedly reported by many research groups [3], and suggested to originate from activation of NADPH oxidase localized in the plasma membrane [4]. The induced generation of H_2_O_2_ (the most stable and widespread form of ROS) plays an important role in the ABA-dependent changes in activity of ion channels bringing about stomatal closure [5]. It can be assumed by analogy that ROS are involved in the regulation of other ABA-induced processes, e.g., an increase in hydraulic conductivity known to be affected by this hormone [6]. It was shown that H_2_O_2_ could function in part as a signal that mediated the ABA induced rise of hydraulic conductivity [7]. Membrane water channels aquaporins (AQPs) have been mentioned in the cited article. However, their involvement in the ROS mediated ABA effects on abundance of AQPs has not been studied.

It was previously shown that up to 80% of the hydraulic conductivity of *Arabidopsis* is determined by the activity of AQPs [8]. ABA influences activity of AQPs at transcriptional level and due to post-translational (phosphorylation) modifications, and this explains its effect on hydraulic conductivity [9]. Peroxide is also known to affect AQPs activity [10]. However, the ABA-induced increase in the activity of AQPs could not be easily related to H_2_O_2_ generation. High concentrations of peroxide reduce AQPs activity by reversible alterations in their structure [11], while its low concentrations lead to an increase in AQP activity [12]. The importance of ROS in ABA-induced changes in AQPs has not been frequently addressed. Several aquaporins have been characterized as H_2_O_2_ channels suggesting that PIP aquaporins (plasma membrane intrinsic protein) facilitate the membrane diffusion of H_2_O_2_ to regulate the stomatal movement [13]. However, the reverse effects of ROS on AQPs as well as their consequences for hydraulic conductance has not been considered in this report. Although it was suggested that ABA-induced stomatal closure in *Arabidopsis* is hydraulically controlled by aquaporins [14], changes in hydraulic conductance were not related to ABA-induced production of H_2_O_2_. Effects of ABA on AQPs abundance have been demonstrated in the study of the influence of this hormone on root hydraulic conductivity [15]. However, in this report, no attention was paid to possible involvement of ROS in ABA-induced changes in either AQPs abundance or hydraulic conductivity in roots.

The dual role of ROS concentrations makes it useful to investigate not only ROS itself, but also ROS manipulators, to reveal the role of hydrogen peroxide for ABA-induced changes in hydraulic conductivity and AQPs activity. This approach has been previously applied to study the involvement of ROS in ABA-triggered stomatal closure and hydraulic conductance. The presence of antioxidants prevented ABA effect on stomatal closure and thereby confirmed the importance of ROS in tested reaction [16]. Similarly, importance of ROS for ABA-induced changes in hydraulic conductivity has been detected in *Phaseolus vulgaris* [7]. We used this approach in our research to discover whether ROS are involved in the stimulatory effect of low ABA concentration on hydraulic conductivity and AQPs abundance in barley plants (high ABA concentrations have been shown to induce opposite effects in *Phaseolus vulgaris* [7]). Antibodies to PIP2;2 have been chosen for this study, since previous experiments involving isoform-specific antibodies raised against HvPIP2;1, HvPIP2;2 and HvPIP2;5 showed the clearest ABA-induced effects in the case of PIP2;2 AQPs [15].

## 2. Results

**Figure 1:**
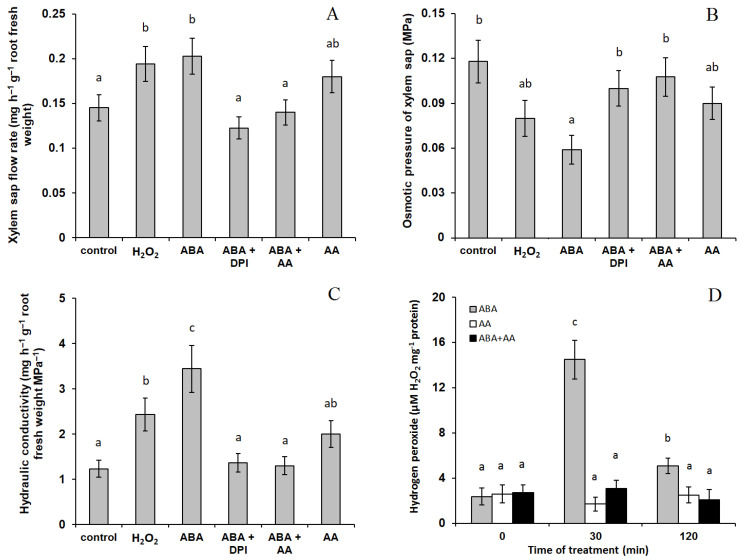
The effect of 10 μM ABA and its combination with 10 μM diphenylene iodonium chloride (ABA + DPI) and 10 mM ascorbic acid (ABA + AA) on the flow rate of xylem sap (**A**), its osmotic pressure (**B**) and hydraulic conductivity (**C**) of detached roots of barley plants; (**D**) production of hydrogen peroxide in roots treated with ABA, AA and ABA + AA. Data represent the mean ± S.E. (*n* = 6). Significantly different means for each variable are labelled with different letters (*p* ≤ 0.05, LSD test).

Immersion of barley roots into the solution containing exogenous ABA led to about 1.5-fold acceleration of the root exudate outflow into the capillary (Figure 1A). The osmotic pressure (OP) of the xylem sap was decreased by abscisic acid as a result of a reduction in the concentration of osmotically active compounds in the sap (Figure 1B). Since the xylem sap OP minus OP of the solution is the denominator in the equation to calculate the hydraulic conductivity, while OP of the solution is close to zero, decrements in the xylem sap OP intensified the difference between the control and the variant with ABA application (3-fold increase in hydraulic conductivity vs. 1.5-fold—For exudates flow) (Figure 1C). Addition of ROS manipulators (ROS scavenger ascorbic acid and a NADPH oxidase inhibitor DPI) simultaneously with ABA prevented the increase in both the flow of xylem sap (Figure 1A) and the hydraulic conductivity of the roots (Figure 1C). The tendency of the increase in the hydraulic conductivity resulting from addition of ascorbic acid alone to the nutrient solution was statistically insignificant.

The presence of hydrogen peroxide in the nutrient medium increased hydraulic conductivity of the roots (Figure 1C), while ABA treatment resulted in increased production of hydrogen peroxide (Figure 1D). Addition of ascorbic acid simultaneously with ABA prevented the increase in hydrogen peroxide production (Figure 1D).

**Figure 2:**
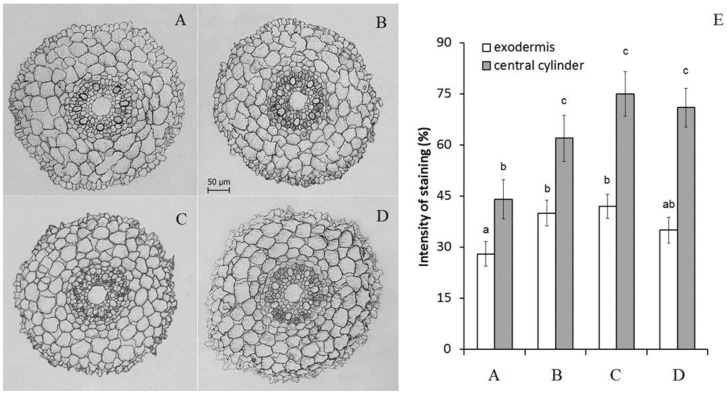
Immunolocalization of ABA (**A**–**D**) and intensity of staining for ABA of exodermis and central cylinder (**E**) (means ± SE, arbitrary units, maximal staining taken as 100%, minimal as 0%) in root sections untreated (**A**), treated with 10 μM ABA (**B**) and its combination with 10 μM diphenylene iodonium chloride (**C**) and 10 mM ascorbic acid (**D**). Data represent the mean ± S.E. (*n* = 50) of arbitrary units; maximal staining was taken for 100%, and minimal staining was 0%. Significantly different means for each variable are labelled with different letters (*p* ≤ 0.05, LSD test).

Immunohistochemical staining revealed an increase in the level of ABA in the roots after the treatment of plants with this phytohormone (Figure 2). These results confirmed that the duration of the hormone treatment was sufficient to increase ABA concentration in the roots due to its uptake. The addition of antioxidants did not change the level of ABA accumulation in the roots.

**Figure 3:**
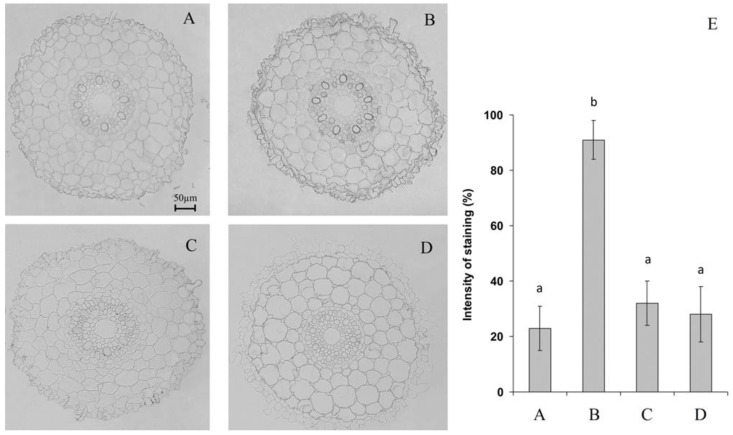
Immunolocalization of PIP2;2 aquaporins (**A**–**D**) and intensity of staining for PIP2;2 aquaporins (**E**) (means ± SE, arbitrary units, maximal staining taken as 100%, minimal as 0%) in root sections untreated (**A**), treated with 10 μM ABA (**B**) and its combination with 10 μM diphenylene iodonium chloride (**C**) and 10 mM ascorbic acid (**D**). Data represent the mean ± S.E. (*n* = 50) of arbitrary units; maximal staining was taken for 100%, and minimal staining was 0%. Significantly different means for each variable are labelled with different letters (*p* ≤ 0.05, LSD test).

The use of antibodies against PIP2;2 revealed an increase in the abundance of these aquaporins under the influence of ABA (Figure 3). The choice of roots for the study of both ABA and AQP localization was dictated by experimental design enabling measurement of hydraulic conductance in detached roots of ABA treated plants. No increase in the level of aquaporins was detected in the roots incubated in the ABA solution combined with either ascorbic acid or DPI (Figure 3).

## 3. Discussion

The ability of ABA to regulate the flow of water from the roots was revealed even earlier than aquaporins were discovered [17]. At that time this phenomenon was explained by the influence of the hormone on the transport of osmotics from the roots, followed with water flow [18]. Our results contradict this point of view, since concentration of osmotics in xylem sap was decreased by ABA due to the dilution in the increased volume of root exudates.

Alternatively, the effect of ABA on the rate of water flow from the roots may be explained by the increase in hydraulic conductivity caused by this hormone due to ABA-induced changes in PIP AQPs located in plasmalemma [7,19]. It is important to note that the method of measuring root hydraulic conductivity applied in the present research allows estimating osmotically driven water flow. In this way, we estimated the cell-to-cell pathway in which AQPs are involved. This assumption is in agreement with obtained results (Figure 1C). Immunolocalization of PIP2;2 in the root absorption zone revealed an increase in the intensity of immunochemical staining of aquaporins in hormone-treated roots (Figure 3B). The abundance of *PIP2*;*2* AQPs transcript as well as that of *PIP2*;*1* and *PIP2*;*5* has been followed in roots and leaves of barley plants and was shown to be high in the roots of barley [20,21]. However, ABA-treatment mostly affected immunolocalization of PIP2;2 protein compared to other PIP2 AQPs [15]. The present data confirmed the role of ABA in the control of abundance of PIP2;2 aquaporins in the root cell membranes as well as roots conductivity in barley plants. ABA is likely to act on PIP2;2 abundance at the post-transcriptional and post-translational levels (e.g., at the level of AQPs trafficking to cell membranes, known to depend on ABA [13]), which may explain the fast response to the ABA application detected in our experiments. Although this hormone is known to influence expression of AQP genes [7,19], this level of regulation is not likely to be involved in such fast responses to ABA-treatment. Importance of ROS-mediated action on AQPs abundance on post-transcriptional and not transcriptional level is supported by the report showing that H_2_O_2_ seems not to be involved in transcriptional regulation of HvPIP2s AQPs in barley [21].

The fact that ABA did not exert its characteristic effect in the presence of ascorbic acid and DPI indicates the necessity of ROS in the transduction of the abscisic acid signal (Figure 3C,D). The results concerning involvement of ROS in ABA-mediated changes in hydraulic conductance are in agreement with the previous data [7], while we were first to show implication of AQPs in the process. The revealed effect of DPI, an inhibitor of flavin-containing enzymes (including NADPH oxidase), is consistent with the data on the capacity of ABA to activate these enzymes responsible for the production of ROS in the apoplast [3].

High concentrations of peroxide rapidly inactivate aquaporins [11]. Our previous study showed inactivation of aquaporins with the Fenton reagent containing peroxide and Fe^3+^ generating ROS in plant tissues in the presence of endogenous peroxidase [22]. However, low ROS concentrations can have an opposite effect, by up-regulation of AQPs genes or modulation of AQPs activity [23]. Therefore, we applied hydrogen peroxide in low concentrations and used an indirect approach, inactivating endogenous peroxide by ascorbic acid or using DPI which inhibited production of peroxide. The obtained results confirmed that ROS are necessary for the action of ABA not only on stomatal conductance, but on hydraulic conductivity. The novelty of our results is in demonstrating that ABA induced changes in PIP2;2 abundance in plasmalemma depend on capacity of plants to produce H_2_O_2_. Revealing exact mechanisms responsible for ABA-induced changes (e.g., possible importance of ROS for the ABA induced trafficking of AQPs to the cell membranes or involvement of AQPs other than HvPIP2;2 in ROS-mediated effect of ABA on hydraulic conductance) should be the goal of further study. The present short communication aims to draw attention of researchers to this problem.

## 4. Materials and Methods

Seedlings of barley *Hordeum vulgare* L. (cv. Prairie) were grown in 0.1 strength Hoagland-Arnon nutrient solution under illumination of 400 µmolm^–2^ s^–1^ at a 14 h photoperiod and 24 °C temperature. Then, 10 μM ABA, 10 mM ascorbic acid or 10 μM diphenyleneiodonium chloride (DPI) were added alone or in combination to the nutrient solution of seven-day-old plants. Concentration of ABA was chosen according to previous experiments, where this concentration was shown to increase hydraulic conductance in barley roots [15]. We used concentrations of AA and DPI recommended by [16], for preventing ABA-induced accumulation of hydrogen peroxide.

To detect hydrogen peroxide (H_2_O_2_) as described [24], 30 and 120 min after addition of ABA and ascorbic acid, barley roots were homogenized in 0.05 M Na phosphate buffer (PB), pH 6.2, at ratio 1:3 (*w*/*v*). Supernatant was separated by 15 min centrifugation at 15,000× *g* in the 5415K centrifuge (Eppendorf, Enfield, United States). The concentration of H_2_O_2_ in the supernatant was assayed using xylenol orange in the presence of Fe^2+^ ions. After staining the mixture, it was centrifuged for 10 min at 8000× *g* followed by reading optical density at 560 nm on the BioSpec Mini spectrophotometer (Shimadzu, Kyoto Japan) and expressed as µM H_2_O_2_ per mg of protein assayed according to Bradford.

After 0.5 h, root hydraulic conductivity was measured as described previously [15,25]. In short, the aerial parts of the plants were removed and roots were connected to capillary by means of silicon tubing. After 1 h, the volume of root exudate was measured by weighing, and the flow rate (J) was calculated as the ratio of the exudate volume to the collection time. The value of J was expressed per unit of root mass. Hydraulic conductivity was calculated as J/(φx − φs), where (φx − φs,) is the difference in osmotic pressure between the root medium and xylem sap measured with the Digital Micro-Osmometer (CAMLAB Limited, Cambridge, United Kingdom).

After root exudate collecting, sections of the root absorption zone identified by the presence of root hairs were fixed with 4% carbodiimide. Dehydrated sections were embedded in the hydrophilic methacrylate resin JB-4 (Electron Microscopy Sciences, Hatfield, PA, USA). Immunolocalization of AQPs and ABA was carried out as described previously [15]. Rabbit sera containing antibodies against aquaporins, or ABA diluted in phosphate buffer with 0.2% gelatin and 0.05% tween-20 were applied to the sections. After incubation for 2 h, the sections were washed three times with phosphate buffer with 0.05% Tween-20. Then, colloidal gold labeled goat immunoglobulins against rabbit antibodies (Aurion, Wageningen, The Netherlands) were applied to each section. After 1 h of incubation, sections were treated with a silver enhancer in accordance with the recommendation of manufacturer (Aurion, The Netherlands). Sections were examined under a light microscope (Carl Zeiss, Jena, Germany) equipped with an AxioCam MRc5 digital camera (Carl Zeiss). Serum containing antibodies against HvPIP2 was kindly provided by Prof. Maki Katsuhara (Institute of Plant Science and Resources, Okayama University, Japan). Antibodies against PIP2;2 were used in this work, since they were mainly influenced by exogenous ABA [15]. The intensity of immunostaining of AQPs and ABA was estimated from 8-bit grayscale images using ImageJ software (National Institutes of Health, Bethesda, MD, USA) as described previously [15]. The intensity of staining was expressed in arbitrary units, with maximal staining taken as 100% and minimal as 0.

Data were expressed as means ± SE, which were calculated in all treatments using MS Excel. Significant differences between means were analyzed by one-way analysis of variance (ANOVA), and a least significance difference (LSD) test to discriminate means.

## Data Availability

Data is contained within the article.
